# Impact of FGF23 level on calcium and phosphorus levels in post-renal transplantation

**DOI:** 10.15171/jrip.2017.19

**Published:** 2016-09-03

**Authors:** Fereshteh Saddadi, Aida Rasoolzadeh, Mohammadreza Ganji, Maryam Miri

**Affiliations:** ^1^Hasheminejad Kidney center Nephrology Ward, Iran University of Medical Sciences, Tehran, Iran; ^2^University of Medical Sciences, Shiraz, Iran; ^3^Department of Nephrology, Shariati Hospital, Tehran University of Medical Sciences, Tehran, Iran; ^4^Kidney Transplantation Complications Research Center, Department of Internal Medicine, Ghaem Hospital, School of Medicine, Mashhad University of Medical Sciences, Mashhad, Ira

**Keywords:** Fibroblast growth factor 23, Kidney transplantion, Phosphorus, Renal function

## Abstract

**Introduction:** The level of fibroblast growth factor 23 (FGF23) may be considered as a prognostic factor for assessing renal function in regulating components of phosphate and vitamin D hemostasis.

**Objectives:** The present study aimed to evaluate the prognostic value of FGF23 level to predict renal function after renal transplantation.

**Patients and Methods:** Fifteen consecutive patients scheduled for renal transplantation. To assess renal function status, the MDRD formula and isotope scan were applied. The study endpoint was to assess the level of FGF23 and other factors involving calcium and phosphorus metabolism before and also 3 and 12 months after transplantation and also to determine role of FGF23 to predict postoperative renal function.

**Results:** The mean level of FGF23 was 839.51±694.56 ρg/mL at baseline that reduced to 44.31±22.01 ρg/mL and 20.13±36.50 ρg/mL, 3 and 12 months after initial assessment. The levels of FGF23 was significantly lower at 3 and 12 months after baseline (*P*=0.01 and *P*=0.02, respectively) with no difference in FGF23 level between the time points of 3 and 12 months after transplantation. Baseline level of FGF23 was found to be higher in the patients with higher glomerular filtration rate (GFR), in older patients, in males, in those patients with diabetic nephropathy, in those with acceptable renal function than in patients who suffered transplant rejection.

**Conclusion:** The level of postoperative FGF23 is an important marker for secretion of phosphorus from kidneys emphasizing the central role of FGF23 marker to regulate calcium and phosphorus metabolism after a successful renal transplantation.

**Implication for health policy/practice/research/medical education:**

The level of postoperative FGF23 is an important marker for secretion of phosphorus from kidneys emphasizing the central role of FGF23 marker to regulate calcium and phosphorus metabolism after a successful renal transplantation.

## Introduction

Fibroblast growth factor 23 (FGF23) as a novel secreted protein hormone encoded by the FGF23 gene is a necessary factor in regulating the phosphate levels and thus phosphate homeostasis (1). This protein is probably secreted by osteocyte in response to elevated phosphate level or other mediators in early chronic kidney disease (CKD) and has many hemostatic roles that maintaining bone strength (2). The regulation of phosphate level is mainly mediated by normal kidney function by stopping reabsorbing phosphate into the bloodstream. This protein can be also released in response to high level of dietary phosphate (3). However conflicting results have been reported in human studies of the effect of dietary phosphorous or calcitriol on serum FGF23 concentrations in healthy individuals.

Recently, significant attention has been focused on FGF23 and its relationship to phosphate homeostasis in patients with renal insufficiency. In some studies, it has been demonstrated that patients with end-stage renal disease have elevated FGF23 concentrations that correlate with serum phosphate concentrations (4). Physiologically, elevations of FGF23 in chronic renal disease likely are due to decreased phosphate clearance as the number of functioning nephrons decrease (5). Due to association between the level of FGF23 and renal functional state especially in those with renal failure, measurement of FGF23 level may provide prognostic information. In dialysis patients with hyperparathyroidism, FGF23 level was more predictive of successful treatment with calcitriol to lower parathormone (6). It has been also revealed that FGF23 concentrations can be significant predictor of refractory hyperparathyroidism (7). It seems that the level of FGF23 can be considered as a prognostic factor for assessing renal function in regulating components of phosphate and vitamin D hemostasis (8), however the value of this marker to predict renal functional status following renal transplantation remained uncertain.

## Objectives

The present study aimed to evaluate the prognostic value of FGF23 level to predict renal function after renal transplantation.

## Patients and Methods

In a prospective study, 15 consecutive patients scheduled for renal transplantation in renal transplantation ward of Shariati hospital in 2013. These patients aged 18 to 65 years had a follow up during the first year of transplantation to handle this study. The exclusion criteria were history of parathyroidectomy, or history of receiving estrogen, progesterone, calcitonin, or bisphosphonate. To assess renal function status, the MDRD formula and isotope scan were applied. A sample of fasting peripheral blood was extracted from all subjects to measure serum levels of calcium, phosphorus, creatinine, intact parathyroid hormone (iPTH), and FGF23 (Human FGF23 intact Elisa, company, Immutopics). The study endpoint was to assess the level of FGF23 and other factors involving calcium and phosphorus metabolism before and also three and 12 months after transplantation and also to determine role of FGF23 to predict postoperative renal function.

### Ethical issues

1) The research followed the tenets of the Declaration of Helsinki; 2) informed consent was obtained, and they were free to leave the study at any time and 3) the research was approved by the ethical committee of Tehran university of medical science by number (ethical code # 92-01-14-20475).

### Data analysis

Results were presented as mean ± standard deviation (SD) for quantitative variables and were summarized by absolute frequencies and percentages for categorical variables. Categorical variables were compared using chi-square test or Fisher’s exact test when more than 20% of cells with expected count of less than 5 were observed. Continuous variables were compared using one-way analysis of *t* test and/or non-parametric Mann-Whitney U test whenever the data did not appear to have normal distribution. All statistical analyses were performed using SPSS software (version 17; SPSS, Chicago, IL, USA). All statistical tests were two-sided, and differences with probability values <0.05 were considered to be statistically significant.

## Results

The mean age of patients was 44.5 ± 13.0 years in male and 39.5 ± 16.0 years in female group. Of those, 73% were male and 26% were female. The underlying renal diseases were glomerulonephritis (n = 3), diabetic nephropathy (n = 3), polycystic kidney (n = 1), and unknown origin (n = 6). Twelve patients received kidney from living donor and others from deceased. The complications of graft rejection and urethral stenosis were observed only in those who received kidney from deceased. Two patients underwent peritoneal dialysis, 12 underwent hemodialysis and one of them was pre-emptive. All study participants were treated with mycophenolate mofetil (1 g/twice daily) 9 with cyclosporine (4-6 mg/kg), and 6 with tacrolimus (0.15-0.3 mg/kg). Except for one patient who developed delayed graft function (DGF) and underwent hemodialysis, other patients had acceptable renal function with mean serum creatinine level of 1.3 ± 0.2 mg/dL and GFR >60 cc/min, in 53.7%. The mean level of FGF23 was 839.51 ± 694.56 ρg/mL at baseline which decreased to 44.31 ± 22.01 ρg/mL and 20.13 ± 36.50 ρg/mL at 3 and 12 months after initial assessment. The level of FGF23 was significantly lower at 3 and 12 months after baseline (*P *= 0.01 and *P *= 0.02, respectively) with no difference in FGF23 level between the time points of 3 and 12 months after transplantation. Baseline FGF23 was significantly higher in the patients with high GFR than in those with low GFR at baseline (*P *< 0.001), however this difference was not significant 3 and 12 months after transplantation (*P *= 0.605). Baseline FGF23 was significantly higher in patients older than 45 years than in the younger at baseline (*P *= 0.001), but this difference was not remained 3 and 12 months after initial evaluation. Furthermore, the level of FGF23 was higher in men than in women at baseline (*P *= 0.03), while there was no difference in FGF23 level at 3 and 12 months between men and women after transplantation. Our study showed that the level of baseline FGF23 level was higher in those patients with diabetic nephropathy (*P *= 0.012), however no difference in the level of this marker between those with and without nephropathy 3 and 12 months after initial assessment. Baseline FGF23 was lower in those patients with serum phosphorus >4.5 mg/dL compared to those with lower phosphorus level (*P *< 0.001), however this discrepancy was not found at 3 and 12 months after baseline. Also, those patients with calcium level <8.5 mg/dL had higher baseline FGF23 level than those with higher calcium level (*P *< 0.001) that was not already significant at 3 and 12 months after. Also, the patients with PTH <150 ρg/dL had higher FGF23 level than those cases with PTH >300 ρg/dL (*P *= 0.002), but this difference was not shown 3 and 12 months after initial assessment. We found that the level of FGF23 was higher in those with acceptable renal function than in patients who suffered transplant rejection (*P *= 0.035). Moreover, those patients who received kidney from living donor had higher FGF23 level than those who received kidney from cadavers (*P *= 0.011) (Table 1).

**Table 1:** Changes in the level of FGF-23 according to baseline factors

**Factor**	**Baseline**	**3 Month later**	**12 Months later**
MDRD			
High>60	928.32 ± 825.82	47.75 ± 19.67	11.95 ± 7.94
Low <60	738.00 ± 554.47	40.39 ± 25.40	29.49 ± 53.32
Gender			
Female	723.90 ± 806.07	34.03 ± 17.66	46.08 ± 69.44
Male	881.55 ± 687.87	48.05 ± 22.95	10.70 ± 7.16
Age group			
> 45 years	1061.16 ± 658.20	47.70 ± 26.21	9.39 ± 5.63
< 45 years	586.19 ± 692.68	40.44 ± 17.21	32.41 ± 52.36
Etiology			
Glomerulonephritis	422.33 ± 341.35	47.63 ± 20.38	10.33 ± 5.65
Diabetic nephropathy	499.50 ± 121.73	23.57 ± 7.92	8.63 ± 7.46
Serum phosphorus			
>4.5 mg/dL	662.60 ± 587.89	48.46 ± 22.16	26.34 ± 46.80
<4.5 mg/dL	1104.87± 810.51	38.10 ± 22.22	10.81 ± 7.19
Serum calcium			
>8.5 mg/dL	645.02 ± 638.63	51.73 ± 20.78	29.01 ± 49.22
<8.5 mg/dL	1061.77 ± 736.02	38.84 ± 21.66	9.98 ± 7.47
Serum PTH			
>300 ρg/dL	769.70 ± 756.38	34.18 ± 20.43	8.36 ± 5.88
<150 ρg/dL	988.95 ± 736.09	51.92 ± 21.54	12.32 ± 7.48
Transplantation outcome			
Acceptable	808.71 ± 687.53	42.57 ± 22.15	10.33 ± 7.08
Rejection	498.00 ± 449.01	65.05 ± 7.70	82.75 ± 95.10
Rocatrol administration			
Yes	503.35 ± 184.79	24.50 ± 8.27	8.57 ± 6.77
No	859.13 ± 805.21	55.42 ± 17.26	30.70 ± 48.61

## Discussion

In our study, the most important finding was 94% reduction in FGF23 level after transplantation compared to baseline level. In fact, the level of this marker reached to normal range in all participants that was similar to the results of Economidou et al (9) findings achieved an 89% reduction in the level of FGF23 after renal transplantation. According to our study, it can be concluded that the patients with preoperative higher level of FGF23 had lower level of serum phosphorus three months after operation. Also, the serum level of calcium reached to normal range that was transplantation similar previous survey (9). In fact, the present study could demonstrate a decreasing in serum phosphorus and an increasing in serum calcium level after transplantation. We also detected that, older adults had significantly higher level of FGF23, which was not reported in previous studies. Likewise, in our higher levels of FGF23 in men than in women was not detected in previous studies. In conclusion it can be explained that successful renal transplantation may lead to considerable decrease in FGF23 level that was significantly associated with decrease in serum phosphorus levels three months after transplantation.

Some previous studies have pointed the role of FGF23 on wasting phosphorus after renal transplantation (9-11). Han et al (10) indicated that FGF23 levels decreased dramatically after renal transplantation. Accordingly Sánchez Fructuoso et al (12) also showed that elevated serum levels of FGF23 could explain the deficiency of calcitriol and elevated renal phosphorus wasting in the early post-transplant period. In another study by Sánchez Fructuoso et al (12), serum FGF23 concentrations remained increased to be elevated in long-term kidney graft recipients, even in the early stages of CKD after transplantation. However, on the contrary, in the study conducted by Tomida et al (13), it has been shown that renal phosphate wasting persisted in the chronic-phase of renal transplantation even with normophosphatemia, however persistent hyperparathyroidism and longer dialysis vintage, but not FGF23, was associated with renal phosphate wasting in the chronic phase.

## Conclusion

The level of postoperative FGF23 is an important marker for secretion of phosphorus from kidneys emphasizing the central role of FGF23 marker to regulate calcium and phosphorus metabolism after a successful renal transplantation.

## Limitations of study

The most important was the small number of the patient and shorter follow up of the patients as a result the association between the level of FGF and renal transplant outcome could not be shown.

## Acknowledgements

The authors thank the Research and Development Center of Shariati Hospital for helping on the statistical aspects of this manuscript**.**

## Authors’ contribution

FS and MM designed the research. AR and MG provided extensive intellectual contribution, collection and analysis of the data and MM and FS wrote some parts of paper. MM prepared the final manuscript

## Conflicts of interest

The authors declared no competing interests.

## Ethical considerations

Ethical issues (including plagiarism, data fabrication, double publication) have been completely observed by the authors.

## Funding/Support

This article is extracted from nephrology fellowship thesis of Maryam Miri. This study was supported by a grant from (Grant #1392, 20475) Tehran University of Medical Sceinces.
